# Rat hepatocytes secrete free oligosaccharides

**DOI:** 10.1016/j.jbc.2024.105712

**Published:** 2024-02-01

**Authors:** Chengcheng Huang, Junichi Seino, Akinobu Honda, Haruhiko Fujihira, Di Wu, Kyohei Okahara, Shinobu Kitazume, Shuichi Nakaya, Ken Kitajima, Chihiro Sato, Tadashi Suzuki

**Affiliations:** 1 Glycometabolic Biochemistry Laboratory, RIKEN-Cluster for Pioneering Research, Wako, Saitama, Japan; 2 Bioscience and Biotechnology Center, Nagoya University, Chikusa, Nagoya, Japan; 3 Institute for Glyco-core Research (iGCORE), Nagoya University, Chikusa, Nagoya, Japan; 4 Discovery Concept Validation Function, KAN Research Institute, Inc, Kobe, Japan; 5 Department of Clinical Laboratory Sciences, School of Health Sciences, Fukushima Medical University, Fukushima, Japan; 6 Analytical & Measuring Instruments Division, Shimadzu Corporation, Kyoto, Japan

**Keywords:** hepatocytes, sialyl free oligosaccharides (glycans), free *N*-glycans, milk oligosaccharides, oligosaccharyltransferase (OST)

## Abstract

We recently established a method for the isolation of serum-free oligosaccharides, and characterized various features of their structures. However, the precise mechanism for how these glycans are formed still remains unclarified. To further investigate the mechanism responsible for these serum glycans, here, we utilized rat primary hepatocytes to examine whether they are able to secrete free glycans. Our findings indicated that a diverse array of free oligosaccharides such as sialyl/neutral free *N*-glycans (FNGs), as well as sialyl lactose/LacNAc-type glycans, were secreted into the culture medium by primary hepatocytes. The structural features of these free glycans in the medium were similar to those isolated from the sera of the same rat. Further evidence suggested that an oligosaccharyltransferase is involved in the release of the serum-free *N*-glycans. Our results indicate that the liver is indeed secreting various types of free glycans directly into the serum.

In recent years, sialyl free oligosaccharides have been identified in the serum as well as in urine samples in human and various animals (1, 2, 3, 4, 5, 6, 7, 8, 9, 10). Changes in some specific structures of sialyl free *N*-glycans (FNGs) have been shown to be related to the growth status of animals, and the overall levels of serum sialyl FNGs has also been shown to be increased in patients with certain types of liver disease (10), implying a close relationship between glycan catabolism and the pathophysiological conditions of the liver. In spite of this, the mechanisms responsible for how these extracellular free glycans reach the serum and how they are correlated with the disease status remains largely unknown.

The catabolic mechanism of intracellular FNGs has been well characterized. In mammalian cells, the major FNGs are released from the lipid-linked precursor for *N*-linked glycoproteins, that is, the dolichol-linked Glc_3_Man_9_GlcNAc_2_, in the endoplasmic reticulum (ER) (11, 12). An oligosaccharyltransferase (OST) is responsible for the hydrolysis of the dolichol-linked glycan in the ER lumen (11, 13, 14, 15, 16) and the released glycans are eventually transported from the ER lumen into the cytosol (17, 18). In the cytosol, an endo-β-*N-*acetylglucosaminidase (ENGase) (19) cleaves a single GlcNAc from the reducing terminal of these Gn2-type glycans which contain *N,N′-*diacetylchitobiose at their reducing termini, and generate Gn1-type glycans bearing a single GlcNAc at their reducing ends. An α-mannosidase 2C1 then further trims the Gn1-type FNGs to produce a Man_5_GlcNAc glycan (20) to facilitate the transport of the glycans into the lysosomes for further degradation (21, 22).

In addition to the glycans from lipid-linked precursors, FNGs could also be released by the cytosolic peptide:*N*-glycanase (Ngly1) (23) from misfolded proteins that are retrotranslocated from the ER to the cytosol (24). The cytosolic Ngly1, ENGase, and α-mannosidase 2C1 constitute a nonlysosomal degradation pathway for high mannose-type FNGs in mammalian cells (25, 26).

In addition to the nonlysosomal glycan degradation pathway, lysosomes have been recognized as the major organelle for the catabolism of *N*-glycoproteins (24). Because of the presence of various catabolic enzymes, complex-type glycans can be fully digested into monosaccharides in the lysosomes under normal conditions, and mutations in those lysosomal enzymes that are involved in glycan catabolism can result in the accumulation of degradation intermediates, thus causing genetic disorders that are collectively referred to as lysosomal storage diseases (27). It has also been shown that Gn1-type sialyl FNGs accumulate in the cytosol of certain cancer cell lines or tissues (28, 29, 30, 31), as well as autophagy-defective cells (32). It has been suggested that these glycans are derived from the degradation intermediates of *N*-glycoproteins in lysosomes, but an in-depth knowledge of the mechanism responsible for their release into the cytosol remain unclarified.

In our previous study, we identified various types of free oligosaccharides in the serum, that is, Gn1 and Gn2-type sialyl FNGs, oligomannose-type FNGs and some sialyl lactose/LacNAc-type glycans in the serum (4). In this study, we used rat primary hepatocytes and compared the structures of the free glycans isolated from the culture medium with those found in the serum of the same rat. As a result, we successfully identified similar structures of sialyl FNGs, oligomannose-type FNGs, as well as sialyl lactose/LacNAc-type glycans from both the serum samples and hepatocyte culture medium. Moreover, NGI-1, a known cellular OST inhibitor (33), significantly reduces the secretion of both the sialyl and oligomannose-type FNGs, suggesting that the OST is, at least partially, involved in the release of FNGs. Our results support our hypothesis that the liver is the origin of the various types of free glycans that are found in serum.

## Results

**Figure 1:**
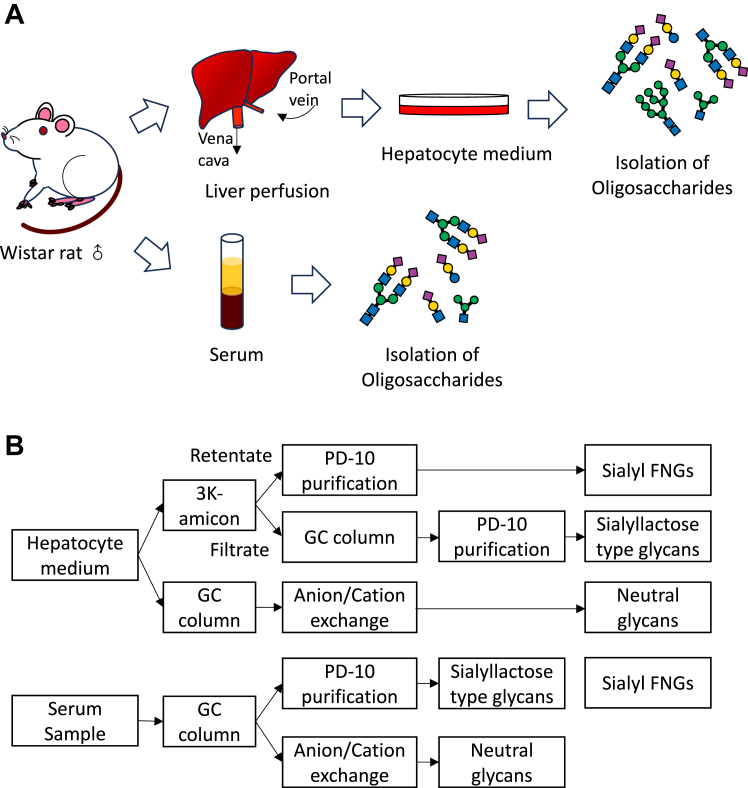
**Flowchart for the isolation of primary hepatocytes and the detection of free glycans in the serum and the medium sample.***A*, scheme of this study. Male Wistar rats of 6 to 8 weeks old were used, and the primary hepatocytes were isolated as described in “Experimental procedures”. The culture medium for primary hepatocytes and serum were collected, and the free glycans were isolated from both the medium and the serum samples for analysis. *B*, flowchart for the isolation of free glycans. *Top*: method for the isolation of free glycans from the hepatocyte medium; *bottom*: method for the isolation of free glycans from serum (4).

FNGs containing sialic acid at the nonreducing terminal end have been characterized in various animal sera (4, 10). We previously noted that there are structural similarities between the structures of the major serum sialyl FNGs with the major *N*-glycans reported on serum glycoproteins; for example, the most abundant sialyl FNG in pigs is a core-fucosylated, NeuAcα2-6-terminated biantennary *N*-glycan (10), which was also identified as the major *N*-glycan structure in serum *N*-glycoproteins (34), most of which are believed to be secreted from the liver. We therefore hypothesized that the liver might also secrete free glycans into the serum in a manner similar to that for the secretion of *N-*glycoproteins. To confirm this hypothesis, we isolated primary hepatocytes from rats and purified free oligosaccharides from the culture medium. The structures of the free glycans that are secreted from the primary hepatocytes were then compared with those found in the serum of the same rat (Fig. 1*A*).

In our previous study, we established a method for isolation of various types of free glycans, that is, sialyl and neutral FNGs, as well as sialyl lactose/LacNAc-type free glycans, from commercially obtained rat serum (4). The established method was applied to the purification of free glycans in serum in this study (Fig. 1*B*, bottom). On the other hand, we found the same procedure used to purify the glycans was not compatible with that for purification of the glycans from culture medium of rat primary hepatocytes, possibly because of the higher salt concentration and the larger sample volume of the medium, leading to substantial contamination by nonspecific peaks after the 2-aminopyridine (PA)-labeling of the free glycans. Thus, after removing the proteins by ethanol precipitation, we separately purified the three different types of glycans based on their characteristics and modified the strategy for isolating the free glycans from the culture medium as follows; the Amicon-based isolation method (9, 10) was applied, and the sialyl FNGs were isolated from the retentate and the sialyl lactose/LacNAc-type glycans in the filtrate faction were further purified using a porous graphite carbon (GC) column (Fig. 1*B*, top); on the other hand, neutral glycans were isolated separately using GC columns, followed by passing the samples through an anion/cation exchange resin to remove the charged materials. This method was originally used for the isolation of neutral FNGs from yeast (35) (Fig. 1*B*, top).

**Figure 2:**
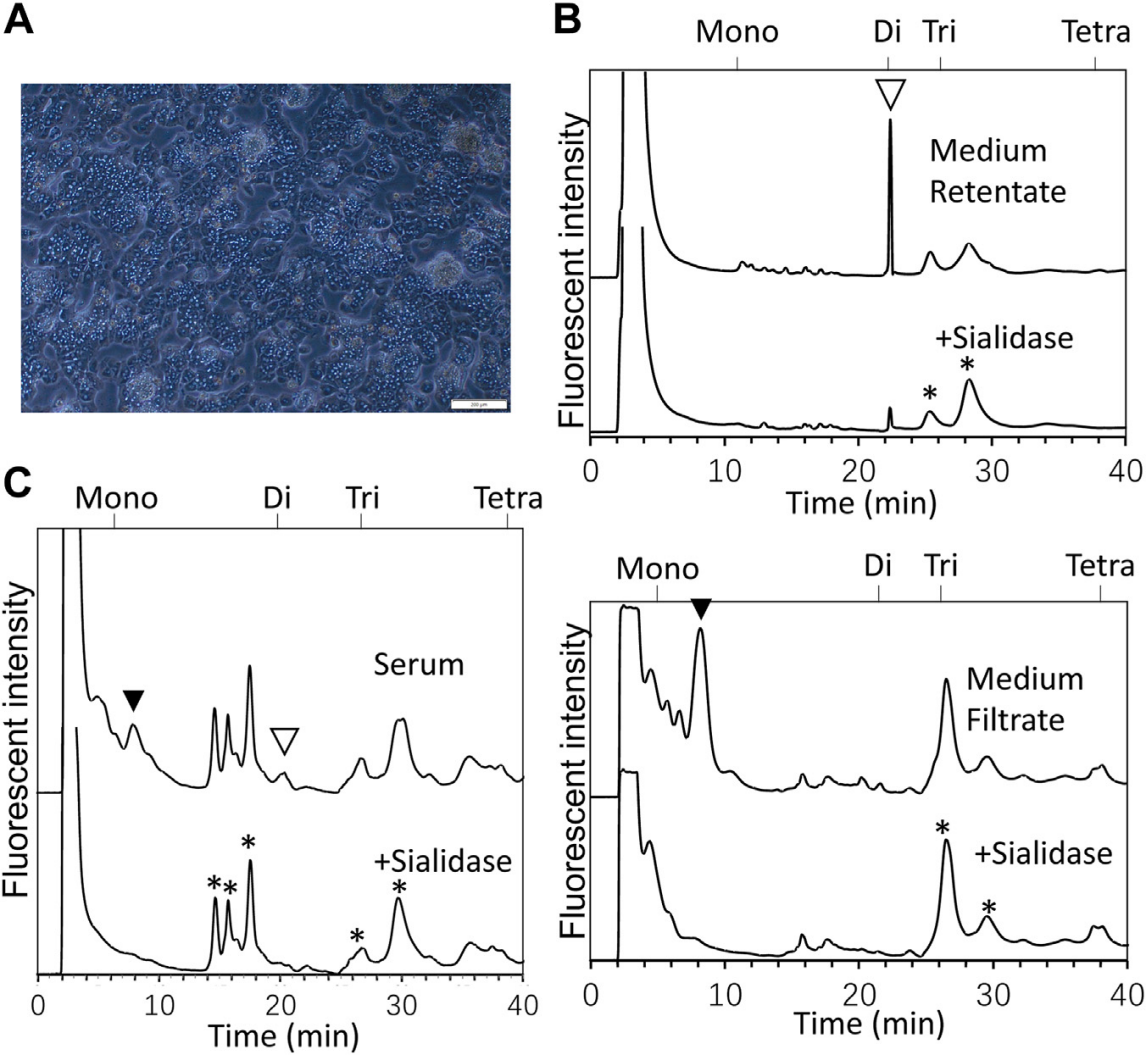
**Sialyl free glycans were detected in both the medium and the serum sample obtained from the same rat.***A*, representative image of the isolated primary hepatocytes (second day culture). The scale bar represents 200 μm. *B*, (*top*) DEAE anion exchange HPLC profile of the free sialyl FNGs (“Medium retentate” fraction) obtained from the second day culture medium of primary hepatocytes, and the sialidase-treated control; (*bottom*) DEAE anion exchange HPLC profile of the sialyl lactose/LacNAc-type free glycans (“Medium filtrate” fraction) obtained from the second day culture medium of primary hepatocytes, and the sialidase-treated control. *C*, DEAE anion exchange HPLC profile of the serum-free glycans obtained from the same rat. *Open arrowhead* indicates the sialidase-sensitive FNGs, and *black arrowhead* indicates the sialyl lactose/LacNAc-type free glycans. *Asterisks* marked in sialidase treated samples in *B* and *C* represent the peaks that are assumed to be nonspecific ones as they are resistant to sialidase. Moreover, no glycan-related signal was detected when the peaks between 12 to 18 min in *C* were collected and subjected for MS analysis. The neutral products are not detected in this HPLC system. DEAE, diethylaminoethyl cellulose; FNGs, free *N*-glycans.

Rat primary hepatocytes were prepared from a 6 to 8 weeks old male Wistar rat (Fig. 2*A*), and the free glycan fractions were isolated both from the hepatocyte culture medium and from the serum of the same rat. To avoid contamination of free glycans from animal sera, chemically defined serum-free medium was used for the culture of the primary hepatocytes. In our experiments, the primary hepatocytes could be maintained for up to 3 days using this serum-free media.

The diethylaminoethyl cellulose (DEAE) anion exchange HPLC profile of the PA-labeled glycans indeed showed sialidase-sensitive peaks in the culture medium of the primary hepatocytes both for the larger sialyl FNG (Amicon rententate) and the small sialyl free glycan (Amicon filtrate). (Fig. 2*B*). It is particularly noteworthy that some of the peaks isolated from the culture medium were found to elute at the same positions as those isolated from the serum of the same rat (Fig. 2, *B* and *C*).

**Figure 3:**
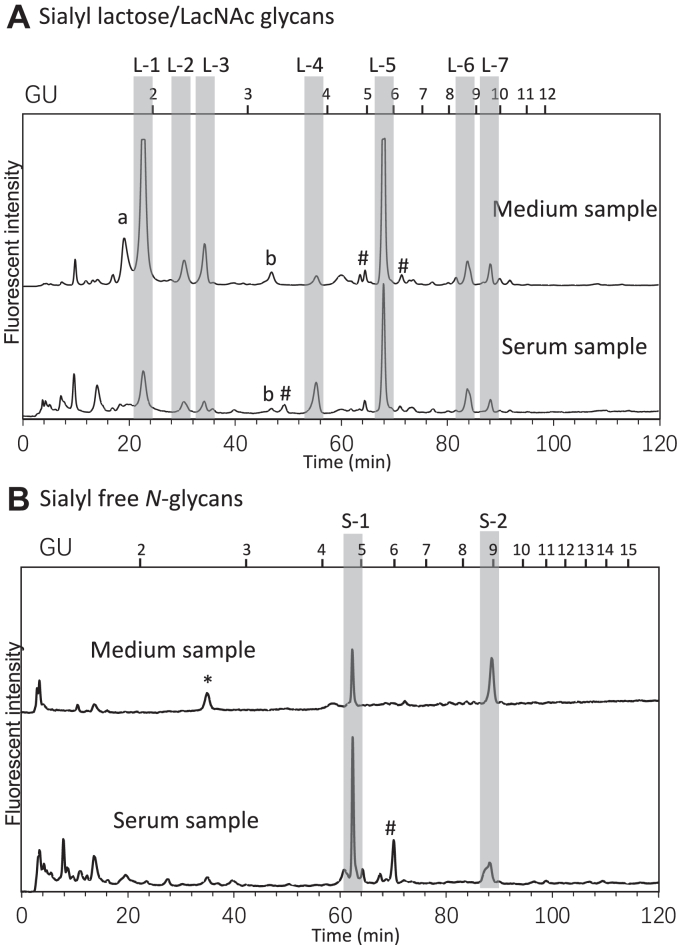
**Sialyl free glycans from serum and medium share same structures.***A*, dual gradient ODS HPLC profile of sialyl lactose/LacNAc-type free glycans collected form medium and serum samples. The sialidase treated control for the fractions was shown in Fig. S1. Peaks indicated by *a* and *b* represent the sialyl glycans whose structures could not be identified by combination of MS, enzyme reactions, as well as their elution positions in HPLC: predicted composition of each peak is as follows: *a*, NeuAc-Hex_2_; *b*, AcO-NeuAc(α2-3)-Hex_2_. Octothorpes represent the minor sialyl small glycans whose structures have not been determined due to its low amount. *B*, dual gradient ODS HPLC of disialyl FNGs collected from the medium and serum samples. L1-7, S1, and S2 marked in gray bars indicate the sialyl glycans detected in the medium and serum samples. *Asterisks* represent the fractions that are shown to be non-sialyl-glycan derived content after the fraction was collected for further examination by enzyme reaction. Octothorpes represent the fractions whose structures have not been identified, as its elution position on a dual gradient ODS HPLC does not match to standard samples available to us. The numbers above the chart are the elution positions of standard glucose oligomers with the number of glucose units (GU). Glycan structures and sugar compositions of L1-7, S1, and S2 were indicated in Table 1. FNGs, free *N*-glycans; ODS, octadecyl-silica.

We fractionated the small sialyl glycans as well as the disialyl glycans from the serum and medium, respectively, and the samples were further separated through a dual gradient octadecyl-silica (ODS) HPLC system (36) (Fig. 3, *A* and *B*). The HPLC profile patterns between the serum sample and the medium sample were very similar (Figs. 3, *A* and *B* and S1*A*). Further analyses clearly indicated that the major free glycans shared the same structure (Table 1). These results suggest that hepatocytes are indeed secreting free glycans into serum, and that these free glycans contain not only the sialyl FNGs but also the sialyl lactose/LacNAc-type free glycans. It should also be noted that the sialic acid residue of the sialyllactose/LacNAc-type glycans were modified by *O*-acetylation, and an *O*-acetylated sialyl lactose unit was one of the most abundant glycans among the sialyl lactose/LacNAc-type glycans that were detected. This modification was identified to be 9-*O*-acetylated NeuAc (Fig. S1*B*). It is also noteworthy that, regarding these sialyl lactose/LacNActype glycans, our previous analyses of commercially available rat sera contained relatively abundant levels of NeuGc (4), but not *O*-acetylated NeuAc. We postulate that such a discrepancy in the modification on the sialic acid may be strain-specific. We also noted the occurrence of some novel, previously unidentified serum sialyl lactose-type glycans, which were determined to be disialyl free glycans with a core lacto-*N*-neohexaose structure, with the NeuAc modified by *O*-acetylation (Fig. S2*A*). The structure was confirmed by digestion with various glycosidases, a reducing-end glycan analysis as well as LC-MS analysis (Fig. S2).

**Table 1:** Predicted structures of sialyl free glycans

Peak	Composition(MS)	Sialidase S (α2-3)	Proposed structure	Notes
L1	NeuAc1Hex2	+	NeuAcα2-3Galβ1-4Glc	a
L2	NeuAc1Hex1	-	NeuAcα2-6Gal	a
L3	NeuAc1Hex1HexNAc1	-	NeuAcα2-6Galβ1-4GlcNAc	a
L4	NeuAc1Hex1HexNAc1	+	NeuAcα2-3Galβ1-4GlcNAc	a
L5	Ac1NeuAc1Hex2	+	AcO-NeuAcα2-3Galβ1-4Glc	a
L6	Ac1NeuAc1Hex1HexNAc1	-	AcO-NeuAcα2-6Galβ1-4GlcNAc	b
L7	Ac1NeuAc1Hex1HexNAc1	+	AcO-NeuAcα2-3Galβ1-4GlcNAc	b
S1	NeuAc2Hex5HexNAc3	-		c
S2	NeuAc2Hex5HexNAc4	-		c

a Reducing end mono/di saccharide was confirmed by AXI anion exchange HPLC.

b The most major peak identified.

c Glycan structure was confirmed by comparing the HPLC profile with previously characterized standard samples.

**Figure 4:**
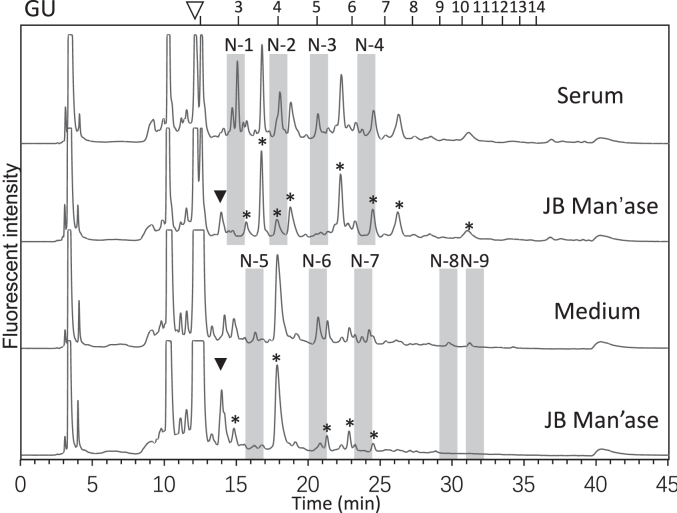
**Size fractionation HPLC profile of oligomannose-type FNGs from the serum and medium samples.** HPLC profiles of the neutral fractions isolated from the serum (*upper*) and the medium (*lower*) samples and the jack bean α-mannosidase (JB Man’ase)-treated controls. *Open arrowhead* indicates the elution position of Man_1_GlcNAc_1_-PA, and *black arrowhead* indicates the elution position of Man_1_GlcNAc_2_-PA. *Asterisks* indicate the peaks that are most possibly non-glycan-derived, as they are resistant to various glycosidase digestions (glucoamylase, JB Man’ase, β-HexNAc’ase, as well as bovine Gal’ase). Peaks N1-9 marked in gray bars indicate the JB Man’ase-sensitive oligomannose-type glycans detected in the medium and serum samples. Sugar compositions indicated in Table 2 were estimated based on the elution positions of the samples on the size fractionation HPLC and the comparison with those of the authentic, previously characterized oligo/high mannose-type glycans (4, 36). The numbers above the chart are the elution positions of standard glucose oligomers with indicated glucose number as glucose unit (GU). FNG, free *N*-glycan.

Since we previously identified neutral glycans in the rat serum samples, we also analyzed the neutral fractions both in the serum and the medium samples. The hepatocyte medium was found to contain abundant level of glucose oligomers, most likely derived from glycogen, and these peaks disappeared after the sample was digested with a glucoamylase enzyme. After the glucoamylase-treatment, the occurrence of α-mannosidase-sensitive, oligomannose-type FNGs was confirmed (Fig. 4). It is intriguing to note that the pattern of the oligomannose-type glycans was different between serum and the medium samples; the most abundant oligomannose-type glycan in the serum turned out to be Man_2_GlcNAc_1_, a result that was consistent with a previous observation (4). On the other hand, the hepatocyte culture medium tended to contain larger glycans that contained Man_8-9_GlcNAc_2_ structures (Fig. 4 and Table 2). It is thus suggested that there might be a system for the catabolism of the oligomannose-type glycans in the blood that would allow the secreted glycans to be demannosylated as the glycans circulate in the bloodstream. Glycosidases, including α-mannosidase, have been reported to occur in the bloodstream of healthy mice and human (37). We detected the activity of α-mannosidase in the rat serum using a *p*-nitrophenyl-α-D-mannopyranoside and indeed found enzyme activity in the serum even at pH around 8 (Fig. S3, *A* and *B*), while the optimal pH for the α-mannosidase in the serum turned out to be around 4.6. Similarly, β-galactosidase (β-Gal’ase) and β-*N*-acetylhexosaminidase (β-HexNAc’ase) were also identified both at pH 4.6 and at physiological pH (Fig. S3*C*). However, we did not detect sialidase activity in the serum using 4-MU-NANA as a substrate (Fig. S3, *D* and *E*). This may be the reason why the sialylated glycans are relatively stable in the animal sera (10).

**Table 2:** Compositions of oligo/high-mannose-type free glycans

Peak	Proposed glycan	Notes
N1	Man_2_GlcNAc	a
N2	Man_3_GlcNAc	a
N3	Man_4_GlcNAc	a
N4	Man_5_GlcNAc	a
N5	Man_2_GlcNAc_2_	a
N6	Man_4_GlcNAc	a
N7	Man_5_GlcNAc	a
N8	Man_8_GlcNAc_2_	ab
N9	Man_9_GlcNAc_2_	ab

a Glycan compositions were deduced based on their sensitivity to JB mannosidase, elution position in size fractionation HPLC and their comparison to known standard samples.

b The GlcNAc_2_ on the reducing end was confirmed based on the formation of ManGlcNAc_2_ on the dual gradient HPLC after the JB mannosidase treatment.

Furthermore, we noted that among oligo/high mannose-type FNGs, there are Gn2-type glycans identified in the media, whereas in the serum, major glycans turned out to be Gn1-type (Fig. 4). Based on our previous studies using mice sera, it has been shown that, although an increase in the level of Gn2-type glycans was obvious in *Engase*^−/−^ mice, over 50% of Gn1-type oligomannose-type glycans were still detected (4), leading us to speculate that lysosomal chitobiase, another enzyme known to convert Gn2-type glycans to Gn1-type ones, may also play a significant role in the release of Gn1-type FNGs. As we did not detect the chitobiase activity at least under the experimental conditions applied (Fig. S3*F*), we do not know how exactly the Gn1-type glycans were generated in the sera in an ENGase-independent fashion.

Based on the above collective results, we concluded that free glycans in the sera are most likely secreted from the hepatocytes. To see whether this phenomenon was also observed in hepatocyte-derived cancer cell lines, we examined the release of free glycans by HepG2 cells. The cells cultured in serum-free medium after seeding, and the sialyl and neutral glycans in the medium were analyzed (Fig. S4, *A* and *B*). We did not find a detectable level of neutral free glycans in the media of HepG2 cells (Fig. S4*B*). On the other hand, the major sialyl glycans turned out to be sialyl lactose/LacNAc-type glycans (Fig. S4, *A* and *C*), suggesting that the formation of sialyl FNGs appeared to be rather unique to primary hepatocytes.

To further investigate specifically how those free glycans are generated in hepatocytes and how they are secreted from the cells, we next investigated which pathway is responsible for the formation of serum-free glycans, especially the sialyl FNGs and oligomannose-type FNGs. Based on our previous studies, it would appear that there are mainly three pathways involved in the release of free *N*-glycans, that is, (1) hydrolysis of the lipid-linked oligosaccharide precursor (11, 13, 14, 15, 16); (2) the cytosolic deglycosylation of *N-*glycoproteins by Ngly1 or ENGase; (3) the oligosaccharides formed as a degradation intermediate in the lysosomes (11). It has been previously shown that Ngly1/ENGase is not likely involved in the formation of major free glycans as there is essentially no difference in the amount of serum FNGs in *Ngly1*^–/–^*Engase*^–/–^ mice (4, 10). Furthermore, lysosomes are unlikely to be the origin as well, since the FNGs predicted to be derived from lysosomes tend to have characteristic structures of degradation intermediates (*i.e.* monosialylated and monoantennary FNGs) (10, 28), while most of the sialyl FNGs found in the sera have intact branch structures/nonreducing end sialic acids.

**Figure 5:**
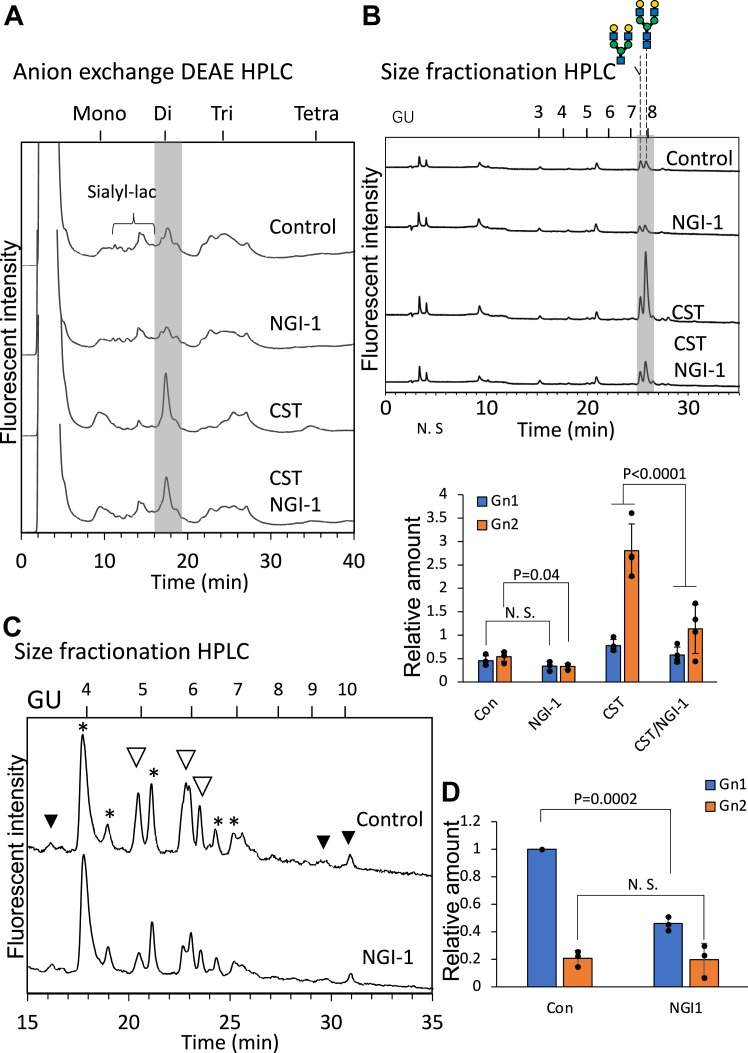
**OST is involved in the release of FNGs into the medium.***A*, DEAE anion exchange chromatography of the sialyl FNGs fractions from the medium of primary hepatocytes. To the medium the following compounds were separately added 1 day after the cells became attached to the dish: DMSO (vehicle control), 5 μM NGI-1(NGI-1), 0.5 mM castanospermine (CST), and 5 μM NGI-1/0.5 mM castanospermine (CST/NGI-1). The inhibitors were incubated overnight and the culture media were collected for free glycan analysis. Peaks marked in gray indicate the disialyl FNGs. *B*, size fractionation HPLC (*upper*) of the major sialyl FNGs in disialylated fractions after sialidase digestion and the calculation of the Gn1/Gn2 ratio (*lower*). The disialylated fractions (marked in *gray* in Fig. 5*A*) were collected, and treated with sialidase before the HPLC analysis. The Gn1+Gn2 glycans in the control was set to 1 and the relative amount in each fraction was calculated. *C*, size fractionation HPLC of the neutral fractions from control (Con) and NGI-1 treated (NGI-1) medium sample. The mannosidase-sensitive peaks that are decreased by NGI-1 treatment are indicated by *open arrowheads*. The mannosidase-sensitive peaks that are not changed after NGI-1 treatment are marked as *black arrowheads*. *Asterisks* indicate the peaks most possibly non-glycan derived, as they are resistant to glucoamylase, JB Man’ase, β-HexNAc’ase, as well as bovine Gal’ase. The samples were fractionated for small glycans (GU<5) and large glycans (GU>5), and further applied to the dual gradient ODS HPLC for the quantification of the change of the glycans after NGI-1 treatment in Fig. S5. *D*, calculation of the relative amount of mannose type FNGs after the NGI-1 treatment. The relative amount of Gn1 and Gn2 type high/oligomannose-type glycans was calculated as a total sum of Man_1_Gn1 and Man_1_Gn2 glycans, respectively, after JB Man’ase treatment as indicated by *black arrowheads* in Fig. S5, and the amount of Gn1 glycans in control sample (without inhibitors) was set to 1. Individual data points were presented, and error bars indicate SD from at least three independent experiments. Two-way ANOVA test was performed for Gn1 and Gn2 glycans to see the influence of inhibitor treatments. The *p* value was calculated by Turkey’s post hoc test, while N.S. represent not significant (*i.e. p* > 0.05). The numbers above the chart are the elution positions of standard glucose oligomers with indicated glucose number as glucose unit (GU). The use of monosaccharide symbols followed the Symbol Nomenclature for Glycans system (73), *blue square*, GlcNAc; *green circle*, Man; *yellow circle*, Gal. DEAE, diethylaminoethyl cellulose; DMSO, dimethyl sulfoxide; FNGs, free *N*-glycans; ODS, octadecyl-silica; OST, oligosaccharyltransferase.

We therefore speculate that lipid-linked oligosaccharides are a major source of serum FNGs, and that OST might be involved in the formation of the serum FNGs. To validate this hypothesis, we treated rat primary hepatocytes with NGI-1, a known cellular inhibitor of OST activity (33). It has previously been shown that this compound inhibits the release of FNGs in *Ngly1*^–/–^*Engase*^–/–^ mouse embryonic fibroblast cells (38). Our results showed that the NGI-1 treatment indeed significantly decreased the amount of Gn2-type sialyl FNGs (Fig. 5, *A* and *B*), as well as the mannose-type FNGs (Figs. 5*C* and S5) in the hepatocyte medium, indicating that OST is involved in the secretion of neutral/sialyl FNGs into the media. It should also be noted that, as for oligomannose-type FNGs, the most significant decrease observed for FNGs by the treatment of NGI-1 turned out to be Gn1-type FNGs. This result indicates that OST is, at least in part, responsible for the formation of these FNGs. We also utilized castanospermine (CST), an α-glucosidase inhibitor, to examine its effect on the secretion of sialyl FNGs of this pathway. The rationale behind this approach is that the removal of glucoses from the Glc_3_Man_9_GlcNAc_2_ has been shown to be important for the efficient translocation of the FNGs into the cytosol for degradation (17, 18, 39). The inhibition of glucose trimming will potentially result in the accumulation of the glucosylated FNGs that can be eventually secreted (40). We however do not believe that is the case, as the Golgi endo-α-mannosidase could bypass the glucose trimming to convert the glucosylated *N*-glycans into complex-type ones (41, 42, 43). Indeed, a previous study showed that treating HepG2 cells with castanospermine impaired the translocation of lumenal FNGs into the cytosol and, accordingly, resulted in the enhanced secretion of sialyl FNGs into the media (40). Consistent with this previous observation, a significant increase in the release of sialyl FNGs by the primary hepatocytes was observed by the CST-treatment, and this increase was also impaired by an NGI-1 cotreatment (Fig. 5, *A* and *B*). Since we identified both the Gn1-and Gn2-type FNGs in the medium and serum samples (4), we next examined the change in Gn1-and Gn2-type FNGs after the treatment with these inhibitors. To this end, we collected the major disialyl FNGs fraction and examined the changes of the ratios after the inhibitor treatment. The findings showed that the NGI-1 treatment did not have a significant impact on the amount of Gn1-type sialyl FNGs. However, a decrease in the Gn2-type sialyl FNGs after NGI-1 treatment was notable, especially when cells were cotreated with CST (Figs. 5*B* and S5). The result is consistent with our speculation that OST is, at least to some extent, involved in the release of the sialyl FNGs from hepatocytes.

## Discussion

In this study, we attempted to clarify the underlying mechanism responsible for the secretion of serum-free glycans, especially sialyl FNGs. Our results clearly demonstrate that hepatocytes secrete various types of free glycans that are structurally similar to those found in sera. Moreover, OST was shown to be involved in the generation of the FNGs. While further studies will be required to clarify the entire pathway for how these glycans are formed, the results reported herein shed light on the mechanism by which these extracellular free glycans are formed.

To date, although there are still some putative transporters/enzymes whose genes remain to be identified, the major process for the intracellular catabolism of FNGs has been well-clarified (24, 25). In sharp contrast, virtually nothing is known regarding the mechanism for how extracellular free glycans are formed. While previous studies indicated that extracellular FNGs in sera or urine may represent novel disease biomarkers (5, 6, 7, 8, 10), our current study suggests that the secretion of various types of serum-free glycans, that is, not only FNGs but also free lactose/LacNAc-type glycans, can be theoretically correlated with the conditions of the liver, from which those glycans are secreted.

Our current study demonstrated that OST is involved in the formation of serum FNGs. Interestingly, our previous study indicated that both cytosolic ENGase and the lysosomal chitobiase appeared to contribute to the generation of Gn1-type oligomannose-type FNGs and the sialyl FNGs (4). The ENGase-dependent generation of Gn1-type FNGs has also been reported in other organisms such as plant (44, 45), or worm (46), and it has been proposed that there may be a retro-translocation mechanism of ENGase-catalyzed Gn1-type free glycans back into the ER. Mechanistic details of such a retrotranslocation system are not available, and accordingly, whether a similar transport system exists in mammalian cells remains to be revealed.

**Figure 6:**
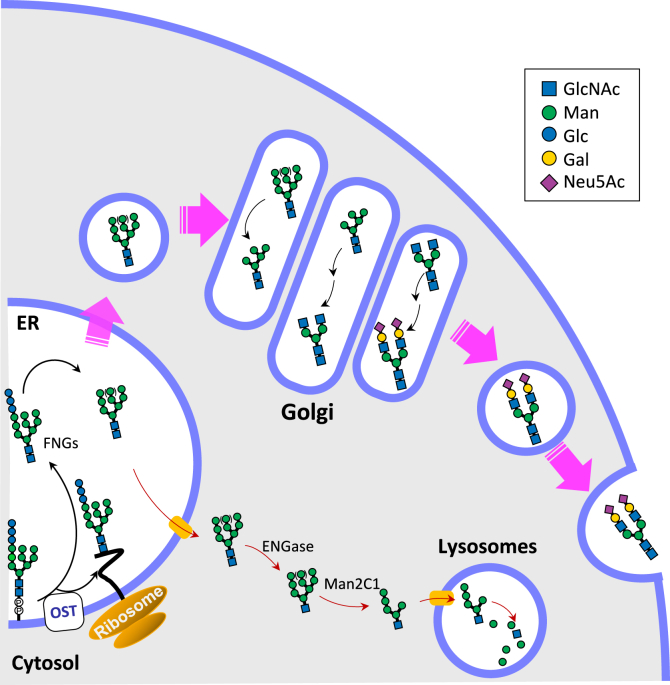
**Proposed model for the secretion of OST-generated Gn2-type FNGs from the hepatocytes into serum.** The free *N*-glycans (FNGs) are released by the hydrolytic activity of OST, and the glycans are transported into the cytosol and processed by cytosolic ENGase and Man2C1, and then transported into lysosomes for further degradation in normal cells (*red arrows*). In hepatocytes, some FNGs escape from the translocation into the cytosol, pass through the vesicular transport and are further processed by the enzymes in the Golgi to convert high-mannose type glycans into complex-type ones. Sialyl FNGs thus formed are eventually secreted outside the cells (*large pink arrows*). The use of monosaccharide symbols followed the Symbol Nomenclature for Glycans system (73), *purple diamond*, NeuAc; *blue square*, GlcNAc; *green circle*, Man; *yellow circle*, Gal; *blue circle*, Glc. ENG, endo-β-*N-*acetylglucosaminidase; FNGs, free *N*-glycans; Man2C1, α-mannosidase 2C1; OST, oligosaccharyltransferase*.*

Based on our results, we formulated a proposed model for the major FNG secretion pathway from hepatocytes (Fig. 6). In mammalian cells, as suggested by previous studies (11, 47), OST constantly releases the Glc_3_Man_9_GlcNAc_2_ into the ER lumen. After the removal of Glc residues, the oligomannose-type glycans are normally transported into the cytosol for further degradation (Fig. 6, bottom, indicated by red arrows). However, in hepatocytes, perhaps due to the enhanced secretion system, a portion of the lumenal FNGs may enter the secretory pathway together with *N*-glycoproteins (Fig. 6, upper, indicated by pink arrows). During the secretion of FNGs, they are modified by glycan processing enzymes and converted from oligomannose-type glycans into sialic acid–containing complex type glycans. It should also be noted that some of the OST-originated, Gn1-type sialyl FNGs appear to be also secreted into the sera (Fig. 5*B*). In our previous study, we also showed that *Engase^^–^/^–^^* mice secreted reduced amounts of Gn1-type sialyl FNGs into their sera, but still significant amount of Gn1-type glycans were observed, indicating that ENGase is only partially involved in the formation of Gn1-type serum FNGs. How exactly these ENGase-generated, or ENGase-independent Gn1-type sialyl FNGs are secreted into the media remains unclarified. We speculate that they may be released either by leakage of intracellular contents from dead cells or through a regulated cellular process equivalent to lysosomal exocytosis or secretory autophagy (48, 49).

It is noteworthy that our results show that the liver can secrete sialyl lactose/LacNAc-type free glycans, whereas milk oligosaccharides have been suggested to be generated exclusively by the mammary gland (50, 51). Similar lactose/LacNAc-type sialyl free glycans have also been identified in human as well as rat milk (52, 53, 54, 55, 56). Lactalbumin, a protein critical for lactose formation in the mammary gland, has not been identified in the liver (57), suggesting that the biosynthesis of lactose/LacNAc-type glycans in the liver may be distinct from the lactalbumin-dependent mechanism that is operative in the mammary gland. We speculate that those lactose/LacNAc-type sialyl free glycans may be synthesized *de novo* from glucose or GlcNAc in the liver. Alternatively, as a distant possibility, it is still possible that at least some of the sialyl lactose/LacNAc structures may theoretically be formed through the action of endoglycosidase/endoglycoceramidase toward glycolipids, while such activities remained to be demonstrated.

To date, it has been suggested that milk oligosaccharides have important biological functions; for example, sialylated lactose type glycans, including the 3/6-sialyllactose, disialyllacto-*N*-hexaose and disialyllacto-*N*-tetraose, which are an important component of human milk, have been reported to reduce the risk for developing necrotizing enterocolitis in neonatal rats and preterm infants (53, 54). Furthermore, there are also reports showing that both an oral and an *in vitro* treatment of 6-sialyllactose modulates the immune reaction and inhibits the secretion of inflammatory cytokines in cases of food allergies (58, 59, 60). As our results indicated that sialyllactose/LacNAc-type glycans that are secreted by rat primary hepatocytes have similar or exactly the same structures as the above-mentioned human milk oligosaccharides (Table 1); it is tempting to speculate that we also benefit from those liver-originated serum glycans in an analogous manner of how infants benefit from milk oligosaccharides in their immune system.

It should also be noted that these liver-produced free glycans may modulate various lectin–glycan interaction events in the blood. It is well known that our immune systems possess various glycan-binding lectins to defend against various crises such as an invasion of pathogenic bacteria. For example, it contains a mannose-binding lectin that initiates the complement system as the first line of immunity (61), and the Siglec family recognizes different sialic acid terminal units in the surface glycoproteins/glycolipid of invading bacteria (62). The serum-free glycans may interact with these lectins to avoid excess immune reactions for foreign peptides or glycans. Furthermore, there are bacterial/viral lectins that efficiently recognize host glycans to their advantage (63, 64, 65). The serum-free glycans released into the bloodstream could therefore play a role in modulating such glycan–lectin interactions, and especially for interactions of bacterial/viral lectins, they could serve as a "decoy" to prevent bacterial/viral infections, and further studies will be required to validate this attractive hypothesis.

## Experimental procedures

### Liver perfusion and isolation of primary hepatocyte

All animal care procedures and experiments conformed to the Association for Assessment and Accreditation of Laboratory Animal Care guidelines and were approved by the Institutional Committee of RIKEN [approval no. H28-2-003(2)].

Primary hepatocytes were isolated following a previously reported method with small modification (66). Briefly, Wistar male rat of 6 to 8 weeks under isoflurane anesthesia (2–2.5% v/v) was opened with a midline incision, the hepatic portal vein was exposed by moving the viscera to the right side of the abdominal cavity, and an 18-Gauge × 1^1^/_4_'' angiocath (Terumo corporation) was inserted into the hepatic portal vein.

After collecting the blood, a 37 °C prewarmed perfusion buffer (Hank’s Balanced salt solution without Ca^2+^ and Mg^2+^, supplemented with 0.9 mM MgCl_2_, 0.5 mM EDTA, and 25 mM Hepes) was connected, and added to the liver though a peristaltic pump. The inferior vena cava was cut to allow efflux, a prewarmed digestion buffer (Hank’s Balanced salt solution with Ca^2+^ and Mg^2+^, supplemented with 25 mM Hepes, 0.05% collagenase (Fujifilm), and a 0.005% trypsin inhibitor (Merck)) was applied after all of the blood was washed away from the liver.

The digestion was stopped when the liver became mushy, and dissected liver was transferred to a 50 ml Falcon tube containing 10 ml chilled serum-free Dulbecco's modified Eagle medium (DMEM) buffer. The cells were then dispersed gently into the medium and passed through an autoclaved 4'' × 4'' cotton gauze and a 100 mm cell strainer (Corning Inc).

The obtained hepatocyte cells were then washed by chilled DMEM (Nacalai Tesque Inc) and collected by centrifuging at 50*g* for 1 min at 4 °C. The cell pellets were then gently resuspended with 20 ml serum-free DMEM. To the sample, 20 ml 90% Percoll solution in PBS(−) was added, followed by a 10 min centrifugation at 200*g*. The cells were then collected at the bottom of the tube and were resuspended in a William’s E medium (Thermo Fisher Scientific) added with 1 × Glutamax (Thermo Fisher Scientific), 50 nM insulin, 50 nM dexamethasone, 100 unit/ml penicillin, and 100 mg/ml streptomycin. The cells were then seeded on a collagen coated 10 cm dish (Iwaki Co, Ltd) at a density of 10^7^ cells/plate. For the inhibitor experiment, the inhibitors indicated were added in the medium on the second day and the medium was collected at the third day of the hepatocyte culture.

### HepG2 cell culture

Mycoplasma-free HepG2 cells were cultured in DMEM supplemented with 10% fetal bovine serum and antibiotics (100 unit/ml penicillin and 100 mg/ml streptomycin) at 37 °C, when the cells are grown into around 70% confluency, the cells were washed twice with William’s E medium (Thermo Fisher Scientific) added with 1 × Glutamax (Thermo Fisher Scientific), 50 nM insulin, 50 nM dexamethasone, and antibiotics, and the cells were cultured in the same medium overnight before the medium was collected for analysis.

### Serum samples

The blood of the rat was collected from the portal vein through the angiocath, and after the clotting at room temperature for 2 h, the serum sample was isolated by a serum separating gel (Sato Kasei) at 5000*g* centrifugation for 15 min at 4 °C.

### Reagents and enzymes

The following enzymes were used for confirming the glycan linkages; sialidase (Neuraminidase, *Arthrobacter ureafaciens*) (Roche Applied Science) specific for α2-3,6,8,9 sialic acid; α2-3-specific sialidase (GLYKO Sialidase S from *Streptococcus pneumoniae*, ProZyme); β1-3,4 galactosidase (bovine) (ProZyme); β 1 to 4,6 galactosidase (jack bean; *Canavalia ensiformis*) (Seikagaku Biobusiness Corporation); α1-2,3,6 mannosidase (*C. ensiformis*) (Seikagaku Biobusiness Corporation); α1-2 mannosidase (*Aspergillus saitoi*) (Agilent Technologies); α1-6 mannosidase (*Xanthomonas manihotis*) (New England Biolabs,); α1-2,3 mannosidase (*X. manihotis*) (New England Biolabs); β-*N*-acetylhexosaminidase (*C. ensiformis*)(Seikagaku Biobusiness Corporation); and α1-2,3,4,6 Fucosidase (bovine) (New England Biolabs).

The colorimetric glycosidase substrates *p*-nitrophenyl-α-D-mannopyranoside (pNP-α-Man), *p*-nitrophenyl *N*-acetyl-β-D-glucosaminide (pNP-β-HexNAc), and *p*-nitrophenyl-ß-D-galactopyranoside (pNP-β-Gal) were from Tokyo Chemical Industry Co., Ltd. The fluorescence-based sialidase substrate 4-Methylumbelliferyl-α-D-*N*-acetylneuraminic acid sodium (4-MU-NANA) was from Sigma-Aldrich.

### Standard PA-labeled oligosaccharides

PA-labeled standard high mannose-type glycans were prepared as reported previously (11, 32, 36). The following PA-labeled glycans were purchased from Takara Bio: PA-sugar chain 026 (Galβ1-4Glc-PA), PA-sugar chain 001 (asialo-biantennary complex-type glycan-PA), PA-sugar chain 023 (α2,6-disialylated biantennary complex-type glycan-PA), The following standard glycans are PA-labeled; NeuAcα2-3-lactose (TCI), NeuAcα2-6-lactose (TCI), NeuAcα2-3-LacNAc (TCI).

### Preparation of free oligosaccharides

Free oligosaccharides were desalted from the serum using a previously established method (4), which also adhere to the minimum information required for a glycomics experiment (MIRAGE) (67). Briefly, to 100 μl serum, 150 μl of ethanol was added to precipitate the proteins. The mixture was incubated on ice for 10 min, and the supernatant after centrifugation at 17,000*g* for 10 min at 4 °C was collected and evaporated to dryness. Samples were dissolved in 0.5 ml water and further purified using an InertSep GC column (GL Sciences; Cat No. 5010–68000), and the free oligosaccharide fraction was eluted with 2.5 ml of 50% acetonitrile containing 20 mM triethylamine-acetic acid (pH 6.0). The fraction was evaporated to dryness, redissolved in 0.5 ml water, and desalted by PD-10 column (GE HealthCare). The desalted samples were dried and subjected to PA-labeling as described previously (35) for HPLC analysis.

Purification of the sialyl free oligosaccharides in the culture medium was performed as follows. Briefly, to 10 ml of medium, 15 ml of ethanol was added to precipitate the proteins. The supernatant after centrifugation at 17,000*g* for 10 min at 4 °C was collected and evaporated to dryness. The resulting sample was then dissolved in 1 ml of water and purified with an InertSepGC column (GL Sciences) as described in the serum glycan purification step. After evaporation to dryness, the eluent was re-dissolved in 0.5 ml of water and then passed through Amicon Ultra 3K-1.5 (Amicon; 3000 molecular weight cutoff) and the low-molecular weight fractions and sialyl FNGs were separated by centrifugation at 10,000*g* for 30 min. The filtration step was repeated 6 times and each time after the filtration, an extra 0.4 ml of water was added to the retentate, mixed, and the filtrate from all steps was collected and mixed as small molecular sialyl glycans-fraction (Sialyllactose/LacNAc-type glycans). After evaporation to dryness, samples were dissolved in 0.5 ml water and further desalted by PD-10 columns (GE HealthCare), dried, and PA labeled for analysis.

Concerning isolation of the neutral glycans from the medium, the eluents from the InertSep GC column were dried, redissolved in 800 μl water and applied onto AG1-X2 (resin volume, 400 μl; 200–400 mesh; acetate form) and AG50-X8 (resin volume, 400 μl; 200–400 mesh; H^+^ form) (Bio-Rad) columns as described previously (35). The resulting flow-through fraction was lyophilized and PA-labeled for HPLC analysis.

### DEAE anion exchange HPLC analysis

For the HPLC analyses of sialyl FNGs, PA-labeled samples were separated by anion exchange HPLC with a TSKgel DEAE-5PW column (7.5 φ × 75 mm; Tosoh) as reported previously (4, 10, 68). The elution was carried out using eluent A (10% acetonitrile containing 0.01% triethylamine) and eluent B (10% acetonitrile, with 7.4% triethylamine and 3% acetic acid) at a flow rate of 1 ml/min at 25 °C, and gradient program was as follows (indicated as % of eluent B): 0 to 5 min, isocratic 0%; 5 to 45 min, 0% to 20%; 45 to 50 min, isocratic 100%; and 50 to 60 min, isocratic 0%. The fluorescence was detected at an emission wavelength of 380 nm with an excitation wavelength of 310 nm. Peaks susceptible to the action of *A. ureafaciens* sialidase (Roche Applied Science) were quantitated. Those sialidase-sensitive peaks were also collected for further structural analyses. Flow through fraction was also collected as neutral glycans for further structural analyses.

### Dual-gradient, reversed phase HPLC analysis

The sialidase-sensitive glycans obtained by anion-exchange HPLC, as well as the neutral glycans, were further separated by a dual gradient revered-phase HPLC analysis using an Inertsil ODS-3 column (2.1 φ × 150 mm; GL Sciences) (36). Briefly, the flow rate was 0.2 ml/min at 25 °C, and eluent A (0.1 M ammonium acetate buffer, pH 6.4) and eluent B (0.1 M ammonium acetate buffer, pH 4.0 with 0.5% 1-butanol) was used; the gradient program (expressed as % eluent B) was as follows: 0 to 10 min, isocratic 1%; 10 to 110 min, 1%-70%; 110.1 to 120 min, isocratic 70%; and 120.1 to 150 min, isocratic 1%. The fluorescence was detected at an emission wavelength of 400 nm with an excitation wavelength of 320 nm. The presence of sialic acid was further confirmed using *A. ureafaciens* sialidase (Roche Applied Science). A 2,3-Specific sialidase (GLYKO Sialidase S from *S. pneumoniae*, ProZyme) was used for the linkage analysis of sialic acid. For neutral glycans, the occurrence of high mannose-type glycans was confirmed by treatment with jack bean α-mannosidase (Seikagaku Kogyo Co). Each fraction was quantitated using the peak area relative to the standard PA-glucose hexamer in the PA-glucose oligomer (2 pmol/μl; Takara Bio, Inc) as a reference.

### Size fractionation HPLC

The neutral glycan fraction in the serum or medium was separated by a size fractionation HPLC with an NH2P-40-3E (3.0 φ × 250 mm; Shodex) column using the following programs: eluent A: 93% acetonitrile in 0.3% acetate buffer (pH 7.0 adjusted by ammonia); eluent B, 20% acetonitrile in 0.3% acetate buffer (pH 7.0 adjusted by ammonia); The flow rate was 0.45 ml/min at 25 °C. The gradient program was as follows: 0 to 0.5 min, 1 to 10% eluent B; 0.5 to 45 min, 10 to 55% eluent B; 45 to 47 min, isocratic 70% eluent B; and 47 to 67 min, isocratic 1% eluent B. The jack bean α-mannosidase-sensitive peaks, collected from dual gradient reversed phase HPLC, were further separated in the same column and eluent A/B using a previously reported program (38).

### AXI anion exchange HPLC for separation of glycans

For the separation of structural isomers of small lactose/LacNAc-type glycans as well as for the determination of reducing end of PA-labeled glycans, a TSKgel Sugar AXI column (4.6 φ × 150 mm; Tosoh Bioscience) was utilized using condition previously described (4). Briefly, isocratic elution with 0.7 M boric acid (pH 9.0 adjusted with potassium hydroxide)/10% acetonitrile was carried out, with a constant flow rate of 0.3 ml/min at 65 °C.

### LC-MS spectrometry

LC-MS analyses were performed according to a previously reported method (4). A Shimadzu LCMS-9030 mass spectrometer coupled with Nexera X2 ultra high-performance liquid chromatograph was used (Shimadzu Corporation). For the separation of glycans, TSKgel Amide-80 (2.0 φ × 150 mm) was used. The mobile phase consisted of 50 mM formic acid/ammonium (pH 4.5) (eluent A) and 100% acetonitrile (eluent B). The LC linear gradient was as follows: 0 to 2 min, 80% eluent B; 2 to 32 min, 80–50% eluent B; 32 to 42 min, isocratic 50% eluent B; 42 to 48.5 min, 50 to 80% eluent B, 48.5 to 63.5 min, isocratic 80% eluent B, with a flow rate of 0.2 ml/min. Samples were injected automatically using SIL-30AC (Shimadzu Corporation), and the injection volume was 45 μl. The column temperature was set to 45 °C. Eluted samples were detected by fluorescence at an emission wavelength of 380 nm with an excitation wavelength of 310 nm.

The mass spectrometer was operated with an electrospray source in the positive ionization mode. The electrospray ionization source conditions were a nebulizer gas rate of 3.0 L/min, a heating gas rate of 10.0 L/min, a drying gas rate of 10.0 L/min, a desolvation line temperature of 250 °C, a heat block temperature of 400 °C, a probe voltage of +4.0 kV, and an interface temperature of 260 °C. MS and tandem mass spectra were obtained using 0.2 s event time. Ar gas was used for collision-induced dissociation. LabSolution software (Shimadzu Corporation) and Shimadzu MS ASSETs Glycan (https://msassets.ms3d.jp/glycan/sign_in) was used for instrument operation and data analysis.

### Analysis of O-acetylated sialic acid residues by DMB derivatization

The samples were hydrolyzed in 0.2 M propionic acid (FUJIFILM Wako Pure Chemical Corporation) by incubating at 80 °C for 4 h. The hydrolysate was dried in a SpeedVac (Savant Instrument, Inc). For 1,2-diamino-4,5-methylenedioxybenzene-2HCl (DMB) derivatization, DMB solution was prepared as described previously (69, 70). Twenty microliters of 0.01 M TFA and 20 μl of DMB solution was added to the dried samples, followed by incubating at 50 °C for 2 h. For the fluorometric HPLC analysis, the reaction mixture was diluted to 10 times and directly injected into a Handy ODS column (4.6 φ × 250 mm; FUJIFILM Wako Pure Chemical Corporation) and eluted with methanol/acetonitrile/0.05% TFA in water (4/6/90, v/v/v). A Jasco LC-900 HPLC system equipped with a Jasco FP-2025plus fluorescence detector (excitation, 373 nm; emission, 448 nm), operating at 1.0 ml/min at a column temperature of 26 °C, was used.

### Serum glycosidase activity assay

The activity of α-mannosidase, β-galactosidase, and β-*N*-acetylhexosaminidase in serum was determined by the established protocol with some modifications (71). Briefly, as for the detection of α-mannosidase activity, to each 30 μl rat serum sample, a final concentration of 10 mM sodium acetate buffer (NaOAc/AcOH, pH 2.6, 3.8, 4.6, and 5.9), 10 mM phosphate-citrate buffer (Na_2_HPO_4_/citric acid, pH 6.8, 7.4, and 8.0) or 10 mM pH8.5 Tris–HCl was utilized to adjust the pH, and 20 μl of 4 mM *p*-nitrophenyl-α-D-mannopyranoside (pNP-α-Man) was then added to start the enzyme reaction. The reaction was carried at 37 °C for 0, 1 h, 2 h, 4 h, or 16 h, and the reaction was stopped by adding the same volume of 500 mM glycine-Na_2_CO_3_ buffer (pH 10.0).

As for comparison for the activity of α-mannosidase, β-galactosidase and β-*N*-acetylhexosaminidase, *p*-nitrophenyl-α-D-mannopyranoside (pNP-α-Man), *p*-nitrophenyl-β-D-galactopyranoside (pNP-β-Gal), or *p*-nitrophenyl *N*-acetyl-β-D-glucosaminide (pNP-β-HexNAc) was utilized, respectively. Serum without any buffer addition was compared with the pH 4.6 sodium acetate buffer-added sample. The reaction was carried out at 37 °C for 16 h and stopped by adding the same volume of 500 mM glycine-Na_2_CO_3_ buffer (pH 10.0). The amount of liberated *p*-nitrophenol was determined by measuring the absorbance at 410 nm using Synergy H1 microreader (BioTek).

The sialidase activity in the serum was determined using 4-MU-NANA according to the previously reported method (72). Briefly, each 5 μl serum was added with 3.5 nmol substrate added with pH 4.6 of sodium acetate buffer to maintain a 10 mM of final concentration, or without adding of buffer to keep a serum normal pH. Sample with only 4-MU-NANA substrate and only serum was utilized as the negative control, and 4-MU-NANA with 0.5 μl of sialidase (*A. ureafaciens*, Roche Applied Science) was added as a positive control. The volume of samples was normalized by water, and the reactions were terminated at indicated time by adding 10 μl of reaction buffer into 100 μl stopping buffer of 0.138 M NaOH in 83% of ethanol solution. The fluorescence was detected using an excitation wavelength of 355 nm and an emission wavelength 460 nm using Synergy H1 microreader (BioTek).

The chitobiase activity in the serum was determined by mixing free-glycans with serum samples. Briefly, free glycans are collected from transferrin by PNGase F (Roche) treatment, the free glycans are then treated with sialidase to remove the sialic acid and further purified with InertSep GC column. The dried sample were then mixed with 100 μl serum at 37 °C overnight, and the reaction was then stopped by addition of 200 μl ethanol, and the free-glycans were purified and PA-labeled for analysis.

## Data availability

All relevant data are contained within this research article and in the supporting information.

## Supporting information

This article contains supporting information (73).

## Conflict of interest

The authors declare that they have no conflicts of interest with the contents of this article.
