# Supplementary material for: Rat hepatocytes secrete free oligosaccharides
Source: J Biol Chem. 2024 Feb 1;300(3):105712. doi: 10.1016/j.jbc.2024.105712 (PMC10912633; doi:10.1016/j.jbc.2024.105712)
Supplement: Supporting information [file mmc1.docx]

**Supporting information**

**Rat hepatocytes secrete free oligosaccharides**

**Chengcheng Huang^1^, Junichi Seino^1^, Akinobu Honda^1^, Haruhiko Fujihira^1^, Di Wu^2,3^, Kyohei Okahara^4^, Shinobu Kitazume^5^, Shuichi Nakaya^6^, Ken Kitajima^2,3^, Chihiro Sato^2,3^, Tadashi Suzuki^1^**

**^1^Glycometabolic Biochemistry Laboratory, RIKEN-Cluster for Pioneering Research, Wako, Saitama 351-0198, Japan;**

**^2^Bioscience and Biotechnology Center, Nagoya University, Chikusa, Nagoya 464-8601, Japan;**

**^3^Institute for Glyco-core Research (iGCORE), Nagoya University, Furo-cho, Chikusa-ku, Nagoya 464-8601, Japan**

**^4^KAN Research Institute, Inc., Kobe 650-0047, Japan;**

**^5^Department of Clinical Laboratory Sciences, School of Health Sciences, Fukushima Medical University, Fukushima, 960-8516, Japan;**

**^6^ Analytical & Measuring Instruments Division, Shimadzu Corporation, Kyoto 604-8511, Japan.**

Supporting information includes Figures S1-5, and one reference paper.

**Figure S1. Identification of 9-*O*-acetyl NeuAc in sialyl lactose/LacNAc-type free glycans. A,** Sensitivity of sialyl lactose/LacNAc-type glycans mixture to sialidase in a dual gradient ODS HPLC. The major peaks Gal, GalGlc (lactose), and GalGlcNAc (LacNAc) were shown in sialidase-treated chart. The shift of the original peaks was indicated by the arrows. **B**, ODS HPLC analysis for sialic acid from L1 and L5 fractions found in small sialyl glycans. The elution positions of standard sialic acids are indicated by open arrowheads. Black arrowheads indicate the NeuAc and 9-*O*-NeuAc detected in the fractions isolated from the medium of rat hepatocytes. Asterisks indicate the non-specific peaks derived from reagents.

**** ****

**Figure S2. Identification of disialyllacto*-N*-neohexaose*.* A,** Structure of disialyllacto*-N*-neohexaose consistently found in medium of rat hepatocytes. **B** to **E**, identification of the core structure. **B,** Size fractionation HPLC of desialylated fraction of disialylated free glycans (**Figure 2B**) collected from culture medium samples from three independent rats. Sialidase-sensitive peak (shown in **Figure 2B**) at the position of disialylated glycan was collected and the samples were treated with sialidase before being applied for the HPLC analysis. The elution of the core glycan was indicated by black arrowheads. **C**, Confirmation of the structure of glycans by glycosidase treatments. (Upper) Desialylated fraction (shown by black arrowhead in **A**) was treated with bovine β1-3,4 galactosidase (β1-3,4 Gal’ase) alone, or treatment of β1-3,4 Gal’ase, followed by a jack bean β-*N*-acetylhexosaminidase (β-HexNAc’ase). Based on the elution position, two Gals were estimated to be removed by β1-3,4 Gal’ase digestion, and two HexNAcs were further removed by β-HexNAc’ase digestion. (Lower) the β1-4 linkage of Gal was further confirmed by the sensitivity towards Jack bean β1-4,6 Gal’ase treatment of the collected fraction indicated by black arrowhead in **B**. **D**, Confirmation of reducing end terminals. The collected fraction was treated with β1-3,4 Gal’ase, boiled and further treated with β-HexNAc’ase. The fraction was further boiled, added with 75% EtOH to precipitate the proteins, and was applied to AXI anion exchange HPLC for structural characterization. The product had the same elution position as the standard PA-lactose. **E**. LC-MS analysis of the desialylated fraction (**Supplemental** **Figure 2B**, indicated by black arrowhead). **F** to **H**, analysis of sialic acid on disialyl lacto*-N*-neohexaose both from the disialyl FNG mixture (**F**, **G**) and the isolated fraction (**H**). **F**, Dual-gradient ODS HPLC of the disialylated fraction mixture from the hepatocyte culturing medium (indicated by open arrowhead in **Figure 2B**). The disialyllacto-*N*-heohexaose was indicated by black arrowhead. **G**, LC-MS analysis of the disialylated fraction mixture from the hepatocyte culturing medium. Upper, total ion chromatogram (TIC), lower, fluorescence chromatogram (Ex 310 nm, Em 380 nm). The disialyl lacto*-N*-neohexaose was marked by black arrowhead. The composition for sialyl FNGs in the sample were shown in the figure, and asterisk indicates non-specific peaks in the sample. **H,** MS chart from LC-MS analysis of the isolated disialyllacto*-N*-neohexaose. Doubly-charged peaks were shown. The use of monosaccharide symbols followed the Symbol Nomenclature for Glycans system (1), purple diamond, NeuAc; blue square, GlcNAc; green circle, Man; yellow circle, Gal; blue circle, Glc. MS data analyzed in 20221020. Responsible person, Chengcheng Huang (RIKEN, hgcgcg@riken.jp).

**Figure S3. Glycosidase activities in the serum. A** and **B**, Detection of α-mannosidase acitiviy using *p*-nitrophenyl-α-D-mannopyranoside (pNP-α-Man). **A**, The activity of α-mannosidase in serum at different pH condition after incubating for different time intervals. **B**, α-mannosidase activity in serum without addition of any buffer from 0-16 h. **C**, Comparison of the serum activity of α-mannosidase (alpha-Man’ase), β-*N*-acetylhexosaminidase (beta-HexNAc’ase) β-Galactosidase (beta-Gal’ase) at pH 4.6 and at serum pH. *p*-nitrophenyl-α-D-mannopyranoside (pNP-α-Man), *p*-nitrophenyl *N*-acetyl-β-D-glucosaminide (pNP-β-HexNAc) or *p*-nitrophenyl-β-D-galactopyranoside (pNP-β-Gal), was utilized for substrates. The reaction was carried for 16 h. **D** and **E**, Detection of sialidase activity in serum using 4-Methylumbelliferyl-α-D-*N*-acetylneuraminic acid sodium (4-MU-NANA). The sialidase activity was detected both at normal serum physiological pH and at pH 4.6, serum and substrate were also incubated separately as negative control. **D,** Fluorescence of the substrate from 1-16 h. We could not detect the increase of the fluorescence more than the baseline (i.e. substrate only+serum only), **E**, Comparison of the fluorescence at 16 h with sialidase containing positive control. **F**, Size fractionation HPLC for the analysis of the serum chitobiase activity on sialidase-treated free Gn2-type glycans that are released from transferrin. The use of monosaccharide symbols followed the Symbol Nomenclature for Glycans system (1), purple diamond, NeuAc; blue square, GlcNAc; green circle, Man; yellow circle, Gal; blue circle, Glc.

**Figure S4. Free glycan analysis on HepG2 culturing medium.** **A** and **B**, sialyl glycans and neutral glycans in HepG2 culturing medium. **A**, DEAE anion exchange HPLC of glycans from HepG2 culturing medium. Sialidase treated sample was analyzed at the same time to identify the sialylated glycans. **B**, Size fractionation HPLC for neutral fraction from the HepG2 medium. The sample amount was increased but no JB Man’ase-sensitive peak was detected. **C**, The peaks of sialyl lactose/LacNAc-type glycans (indicated by white arrows in **A**) were collected for further analysis.

**Figure S5. Change in the Gn1 and Gn2 type glycans after an OST inhibitor NGI-1 treatment.** Dual gradient ODS HPLC of oligomannose-type glycan and the JB mannosidase treated control (**A**, GU<5, 12 min to 20 min in **Figure 5B**), and large mannose-type glycan and the JB mannosidase treated control (**B**, GU>5, 20 min to 35 min in **Figure 5B**). The sample was collected from the size fractionation HPLC of neutral fractions in the medium sample of primary hepatocytes (**Figure 5B**). The black arrowheads indicated the elution positions of Man_1_GlcNAc_1_ and Man_1_GlcNAc_2_, and they were quantified after Man’ase treatment as a total sum of oligomannose-type Gn1 and Gn2 FNGs, respectively, and the results were presented in **Figure 5D**. The use of monosaccharide symbols followed the Symbol Nomenclature for Glycans system (1), blue square, GlcNAc; green circle, Man.

**SI References**

1. Varki, A., Cummings, R. D., Aebi, M., Packer, N. H., Seeberger, P. H., Esko, J. D. *et al.* (2015) Symbol Nomenclature for Graphical Representations of Glycans Glycobiology **25**, 1323-1324 10.1093/glycob/cwv091
